# The pharmacokinetics of dexmedetomidine during long-term infusion in critically ill pediatric patients. A Bayesian approach with informative priors

**DOI:** 10.1007/s10928-016-9474-0

**Published:** 2016-05-24

**Authors:** Paweł Wiczling, Alicja Bartkowska-Śniatkowska, Oliwia Szerkus, Danuta Siluk, Jowita Rosada-Kurasińska, Justyna Warzybok, Agnieszka Borsuk, Roman Kaliszan, Edmund Grześkowiak, Agnieszka Bienert

**Affiliations:** Department of Pediatric Anesthesiology and Intensive Therapy, Poznan University of Medical Sciences, Szpitalna Street 27/33, 60572 Poznan, Poland; Department of Clinical Pharmacy and Biopharmacy, Poznan University of Medical Sciences, Poznan, Poland; Department of Biopharmaceutics and Pharmacodynamics, Medical University of Gdansk, Gdansk, Poland

**Keywords:** Dexmedetomidine, WinBUGS, Population pharmacokinetics, Informative priors

## Abstract

The purpose of this study was to assess the pharmacokinetics of dexmedetomidine in the ICU settings during the prolonged infusion and to compare it with the existing literature data using the Bayesian population modeling with literature-based informative priors. Thirty-eight patients were included in the analysis with concentration measurements obtained at two occasions: first from 0 to 24 h after infusion initiation and second from 0 to 8 h after infusion end. Data analysis was conducted using WinBUGS software. The prior information on dexmedetomidine pharmacokinetics was elicited from the literature study pooling results from a relatively large group of 95 children. A two compartment PK model, with allometrically scaled parameters, maturation of clearance and t-student residual distribution on a log-scale was used to describe the data. The incorporation of time-dependent (different between two occasions) PK parameters improved the model. It was observed that volume of distribution is 1.5-fold higher during the second occasion. There was also an evidence of increased (1.3-fold) clearance for the second occasion with posterior probability equal to 62 %. This work demonstrated the usefulness of Bayesian modeling with informative priors in analyzing pharmacokinetic data and comparing it with existing literature knowledge.

**Electronic supplementary material:**

The online version of this article (doi:10.1007/s10928-016-9474-0) contains supplementary material, which is available to authorized users.

## Introduction

The dosage of most drugs in children is based on extrapolation of pharmacokinetic and pharmacodynamic data obtained from adults using body weight scaling, age and occasionally other patient’s characteristics, such us gene polymorphism [1]. Without taking into account the degree of maturation of various organ in children and neonates in pharmacokinetic extrapolations, over or under dosing might occur, which consequently might lead to serious complications, side effects and lack of expected therapeutic effects [2, 3]. Therefore, the identification of inter-individual differences directly or indirectly affecting pharmacokinetics (PK) of drugs, is very important for selecting the individual and the optimal dose, especially in children under severe conditions. It especially applies to new drugs such as dexmedetomidine (DEX), for which there is a relatively small number of studies performed on special population, like that from pediatric intensive care units (PICU).

DEX as a potent, highly selective and more specific α2-adrenoceptor agonist [4] has become an interesting alternative drug for so far widely used benzodiazepines during general anaesthesia and sedation in intensive care. Its unique characteristics makes it an α-adrenoceptor agonist with α2:α1 selectivity ratio of 1600:1, especially for the α2A subtype, providing increased sedation, anxiolysis and analgesia without breathing depression. Dexmedetomidine is metabolized in liver with hepatic extraction ratio of 0.71 and the mean elimination half-life of about 2–2.5 h. Glucuronidation is the process, which poses one-third of metabolism. The other pathways involve multiple cytochrome P450 enzymes, especially CYP2A6, but also CYP1A2, CYP2C19, CYP2D6, and CYP2E1. Approximately 90 % of administered human drug dose is excreted as metabolites in urine, and 10 % in feces [5]. Its influence on brain and spinal cord, mainly via locus coreuleus (LC), provides effects, which are different from those produced by other standard drugs (e.g. clonidine). It diminishes impaired sleep deprivation or poor sleep quality, especially during long-term sedation. DEX, in contrast to benzodiazepines, does not disrupt REM sleep and more closely resembles natural non-REM phase, as well as regulates the circadian rhythm by shifting sleep from day to the night [6–10]. DEX has been effectively used in patients more often presenting agitation and developing higher risk of delirium during conventional sedation, allowing shortening of mechanical ventilation and thus, stay in the PICU [11–14]. Despite increasing number of clinical experiences, pharmacokinetic and pharmacodynamic characteristics of DEX still remain unclear, forcing the need for further research, especially in the youngest patients [15, 16].

The data obtained from routine clinical monitoring are challenging in terms of interpretation and are often collected in not perfectly-controlled experiments. For such data, using a full conditional Bayesian modeling approach with prior’s information is very appealing. In this work we explore the use of informative priors to analyze the data obtained during routine hospitalization of children in an intensive care unit and to identify differences between our study and the currently established knowledge on DEX pharmacokinetics. The analysis consisted of several steps (1) elucidation of prior’s information on the type of model and its parameters from the literature, (2) development of a pharmacokinetic model, (3) determination of covariate relationship which could explain inter-individual and intra-individual differences in drug PK, and (4) identification of differences between literature-described patients and those enrolled in this study.

The possibility of using informative priors is a particular strength of the Bayesian framework [17]. During this type of analysis the priors and the newly collected data are appropriately weighted yielding a posteriori distribution of parameters and predictions that provide logically consistent inference conditional on all the explicitly stated assumptions, such as structural model and priors. Nevertheless, the Bayesian inference using Markov chain Monte Carlo (MCMC) algorithm is not very popular, as generally it is computer intensive. There are only few population pharmacokinetic analyses published which used WinBUGS [18–22]. Informative prior (with relatively high precision) was rarely used [23–25].

## Materials and methods

### Patients

In our study, DEX was used in addition to the standard algorithm of sedation applicable in our PICU which consists of sufentanyl and midazolam administration [26, 27]. Similarly, sedation monitoring was also carried out by the Cook Scale, which has been routinely used by experienced and trained nurses’ team in our department [28]. This scale was originally adapted from Glasgow Coma Scale (GCS) based on the assessment of four reactions, such as eye opening, cough reflex, respiration and motor activity, in response to the stimulus, ranging from minimum of 4 (deep sedation) to maximum of 18 points (awakening). Decision on the addition of DEX to the standard sedation and analgesia was made by a doctor (paediatric intensivist) in order to prevent delirium and/or facilitate awakening of patient. The pediatric risk of mortality (PRISM) score was determined for all patients in the admission to PICU. It is a physiologically based score used to quantify physiologic status, and when combined with other independent variables, it can compute expected mortality risk and expected morbidity risk [29].

Thirty-eight patients were enrolled in the study. Informed consent was obtained from the parents or legal representatives according to the approval of the Institutional Bioethics Committee (no 276/12). Exclusion criteria included the following factors: age >18 years, known allergy to DEX, previous administration of neuromuscular blocking agents and severe renal and/or hepatic insufficiency with serum bilirubin and creatinine levels twofold higher than upper limits of normal reference values.

Continuous intravenous infusion of DEX was routinely initiated at the rate of 0.8 µg/kg/h. Among the patients requiring mechanical ventilation, infusion of DEX was gradually increased or decreased by 0.2 µg/kg/h to maintain the level of sedation between 7 and 14 points in the Cook Scale. Maximum dose of DEX was 1.4 µg/kg/h. Otherwise, if the doctor decides that the patient could be awakened, DEX was decreased by 0.2 µg/kg/h to its minimum dose, till the end of infusion. At the same time, the doses of sufentanyl and midazolam were alternatively reduced to 0.01–0.05 µg/kg/h and 0.01–0.1 mg/kg/h, respectively, to obtain adequate sedation while maintaining spontaneous respiration. Sedation for each patient included in the study was adjusted individually considering the clinical criteria of intensive therapy.

Blood samples for PK assessment (2.0 mL) were collected from the arterial catheter according to the protocol of the study. The first blood sample was collected just before the initiation of DEX infusion, and further samples were collected at 1, 4, 8, 12, 16, 20, and 24 h during the first day (occasion 1). When DEX infusion was stopped, blood samples were collected just before the cessation, and then, at 5, 10, 20 min and 1, 2, 4 and 6 h after the infusion end (occasion 2). All blood samples were centrifuged immediately after collection, and plasma was stored at −80 °C until analysis.

### Analytical methods

Analytical method description was presented in detail elsewhere [30]. Briefly, extraction of DEX from 500 µl plasma was performed with the use of solid-phase extraction Bond-Elut Plexa cartridges (30 mg, 1 ml, Agilent Technologies, Inc., Palo Alto, CA, USA). Extracted samples were evaporated to dryness at a miVac Quattro Sample Concentrator (Genevac, Suffolk, UK), reconstituted with 100 µl of methanol, and injected onto the chromatographic system. Analyses were performed with the use of an 1260 HPLC system (Agilent Technologies, Inc., Palo Alto, CA, USA) composed of degasser (G1322A), binary pump (G1312B0, thermostated autosampler (G1329B) coupled with triple-quadrupole mass spectrometer (6430) with electrospray ionization source (ESI). The separation was carried out using a Zorbax Eclipse Plus C18 (4.6 × 100 mm, 3.5 µm, Agilent Technologies). The mobile phase, pumped at a flow rate of 0.5 ml/min, was composed of a mixture of water and methanol with addition of 0.1 formic acid (2:8, v/v). The analyses were performed with the use of detomidine as an internal standard (IS). The total analysis time was 3 min.

The software used for data acquisition and processing was MassHunter Workstation v. B.07.01. (Agilent Technologies, Inc., Palo Alto, CA, USA). Ions were detected using multiple reaction monitoring (MRM) acquisition mode at the following mass transitions: m/z 201 → 95 (quantifier), m/z 201 → 68 (qualifier) for DEX and m/z 187 → 81 for IS. The quantification of the analyte concentration was based on area peak ratios of DEX over IS. Mass spectrometry parameters: fragmentor voltage, collision energies and ESI parameters (gas flow, nebulizer pressure, drying gas temperature and capillary voltage) are listed in supplementary material.

The developed and optimized method was validated following the guidelines of the United States Food and Drug Administration (FDA) for bioanalytical method validation [31]. It was validated in terms of linearity, specificity, lower limit of quantification, recovery, intra- and inter-day precision and accuracy, analyte stability during the sample processing and storage as well as in terms of matrix effects; all the parameters met the FDA bioanalytical requirements. Each analytical sequence included double blank sample, blank sample, calibration standards (5, 10, 50, 100, 500, 1000, 2500 ng/ml) and quality controls (20, 200, 2000 ng/ml). The intra- and inter-day precision ranged between 5 and 7.4 RSD, respectively, and accuracy of the assay reached an average of 101.6 and 103.0, for intra- and inter-day tests. LOD, based on S/N ratio 3, equaled 1.5 pg/ml.

### PK model development

Population modeling was performed using WinBUGS 1.4.3. The BUGS language interface was implemented using WBDev and BlackBox 1.5 compiler as described elsewhere [32]. Data management, launching WinBUGS, and analysis of the MCMC samples were done in Matlab Software (Version 8.1; The MathWorks, Natick, MA, USA) using the MatBUGS interface. Three MCMC chains of 6000 iterations were simulated. The first 3000 iterations of each chain were discarded and every 3rd sample was retained. Thus 3000 MCMC samples were used for subsequent analyses. Model convergence was assessed by Gelman-Rubin diagnostics available in WinBUGS. The MCMC chains were assumed to have reached the stationary distribution if Gelman-Rubin values were less than 1.2 for all parameters. Furthermore, the trace history of MCMC samples for all chains was examined visually for all parameters, for which ‘fuzzy caterpillar’ suggests that MCMC chains had reached a stationary distribution [17]. All the codes are available in the Supplementary Materials. Model selection was based on deviance information criterium (DIC), which is the mean of the deviance distribution (−2 log likelihood) plus penalty for the effective number of parameters in the model.

The DEX plasma concentrations were characterized by a two-compartment model. The following equation were used:

1 $$V_{p} \frac{{dC_{p} }}{dt} = R{}_{0}(t) - CLC_{p} - QC_{p} + QC_{T} \quad C_{p} (0) = 0$$

2 $$V_{T} \frac{{dC_{T} }}{dt} = QC_{P} - QC_{T} \quad C_{T} (0) = 0$$

where *C*_*P*_, *C*_*T*_ denotes concentrations of DEX in central and peripheral compartments. The model was parameterized with volume and clearance terms. The *V*_*P*_, *V*_*T*_ denote volumes of distribution of the respective compartments, *CL* denotes metabolic clearance of DEX and *Q* denotes the inter-compartmental clearance. The *R*_0_ denotes the infusion rate and all extra boluses that were administered to a patient. All tested models were parameterized in terms of the natural log of the parameter values (i.e. ln (*CL*)).

Inter-individual variability (IIV) for all PK parameters was modeled assuming log normal distribution:

3 $$\ln P_{i} = \ln \theta_{P} + \eta_{P,i}$$

where *P*_*i*_ are PK parameter for the *i*th subjects, *θ*_*P*_ is the typical value of this parameter in the population, and *η*_*P*_ is a random effect for that parameter with mean 0 and variance *ω*_*P*_^2^.

Any *j*th observation of DEX concentration for the *i*th individual, *C*_*Pij*_ at time *t*_*j*_, was defined on a log scale by:

4 $$\log C_{P,ij} = \ln C_{P} + \varepsilon_{C,ij}$$

where *C*_*P*_ is defined by the basic structural model (Eq. (1)) and represents the additive (on a log scale) random error for PK measurements. It was assumed that is t-distributed with mean 0 and scale of the t-distribution denoted by *σ*_*C*_ and degrees of freedom (or normality parameter) ν to account for some outlying measurements present in the dataset.

The effect of body size on all the volume (*V*_*C*_, *V*_*T*_) and clearance (*CL, Q*) parameters was included based on allometric scaling as follows:

5 $$\ln P_{i} = \ln \theta_{p} + \ln f_{P} + K\ln \left[ {\frac{{BW_{i} }}{70}} \right] + \eta_{P,i}$$

where *P*_*i*_ denotes the individual value of volume and clearance term; the population estimates of volume and clearance terms, *BW*_*i*_ the individual body weight, where 70 is a typical body weight of adult patients, and *K* is the exponent equal to 0.75 for clearance and 1 for distribution volumes [33]. All parameters were different between occasions with a fractional change *f*_*P*_ for occasion 2 (*f*_*P*_ = 1 for occasion 1). Additionally, for clearance an age-dependent maturation was included:

6 $$\ln CL_{i} = \ln \theta_{CL} + \ln f_{P} + K\ln \left[ {\frac{{BW_{i} }}{70}} \right] + \ln \left[ {\frac{{PMA_{i}^{\text{Hill}} }}{{PMA_{i}^{\text{Hill}} + TE_{50}^{\text{Hill}} }}} \right] + \eta_{CL,i}$$

where *CL*_*i*_ denotes the individual value of clearance; *PMA*_*i*_ the individual postmenstrual age of the patient; *TE*_50_, and Hill are Hill equation parameter reflecting the slope and the degree of clearance maturation. Following the WinBUGS parameterization [17, 23] (uncertainty is described as a precision, which is an inverse of variance), the stochastic parts of the model can be represented as:

7 $$\varepsilon_{ij} \sim t(0,\sigma_{C}^{ - 2} ,v)$$

8 $$\log P_{i} \sim {\text{MVN}}(\ln \theta_{P} ,\varOmega^{ - 1} )$$

where t denotes t-distribution and MNV is multivariate normal distribution. The model for the priors is as follows:

9 $$\sigma_{C} \sim {\text{Uniform}}(0.001,1000)$$

10 $$\upsilon \sim 1 + {\text{Exponential}}(0.1)$$

11 $$\log \theta \sim {\text{MVN}}(\ln \bar{\mu },\sum^{ - 1} )$$

12 $$\varOmega^{ - 1} \sim {\text{Wishart}}(\rho \varOmega_{0} ,\rho )$$

The priors consisted of the vector of hyperprior population mean parameters,$\overline{{{{\mu }}}}$, its precision, the expected intra-subject variance Ω_0_ and its precision given by Wishart distribution degrees of freedom ρ. For the residual error model sigma (scale of the t-distribution) was assumed to follow a uniform distribution and ν (normality parameter) was constrained to be larger than one and following an exponential distribution.

### Prior selection

The informative priors for the typical value of PK parameters and their inter-individual variability were elucidated from the work [34] and are presented in Table 1. The priors were obtained from the pooled analysis of four studies investigating DEX pharmacokinetics after i.v. administration to 95 children.

**Table 1:** Prior distributions for *θ* and *Ω* as derived from [34]

Parameter, units	Description	Reported			Used in WinBUGS			
		${\bar{{\mu }}}$	% SE	95 % CI	ln 95 % CI	Log ${\bar{{\mu }}}$	Σ for ln ${\bar{{\mu }}}$ ^a^	Σ^−1^ for ln ${\bar{{\mu }}}$
Fixed effects								
CL, L/h 70 kg^−1^	Total clearance	42.1	4.4	(38.7–45.8)	(3.7–3.8)	3.7	0.00185	542
Q, L/h 70 kg^−1^	Distribution clearance	78.3	14.4	(50.7–98.4)	(3.9–4.6)	4.3	0.02862	34.9
V_1_, L 70 kg^−1^	Volume of distribution of central compartment	56.3	8.7	(44.5–67.4)	(3.8–4.2)	4.0	0.01122	89.2
V_2_, L 70 kg^−1^	Volume of distribution of peripheral compartment	69	8.2	(57.5–80.3)	(4.1–4.4)	4.2	0.00726	138
TE_50_, weeks	Age at which clearance is 50 % of adult value	44.5	6.9	(36.8–50.3)	(3.6–3.9)	3.8	0.00636	157
Hill	Slope of clearance maturation	2.56	17.6	(1.65–3.78)	(0.5–1.3)	0.9	0.04472	22.4

Parameter, units	Description	Reported	Used in WinBUGS	
		% CV	Ω_0_^b^	Ω_0_^−1^
Between subject variability (diagonal elements)				
CL	Variability for CL	30.9	0.091	11.0
Q	Variability for Q	37	0.13	7.79
V_1_	Variability for V_1_	61.3	0.32	3.13
V_2_	Variability for V_2_	47	0.20	5.01
			ρ^c^	30

For Σ, Σ^−1^, Ω_0,_ and Ω_0_^−1^ only diagonal elements are provided (the off-diagonal elements are zero)

^a^Calculated based on (97.5th–2.5th)/2/1.96, where 2.5th and 97.5th are 95 % confidence intervals (on a log scale) from bootstrap

^b^Calculated based on ln((%CV/100)^2 + 1^)

^c^The value of ρ was determined empirically for the variance–covariance matrix by simulating from the Wishart distribution in MATLAB (Version 6.5, The MathWorks, Natick, MA) to ensure 25 % of variability in Ω_0_ parameters. The standard errors for %CV were not reported in the original article

### Covariates

The potential effect of various covariates (listed in Table 2) on DEX PK was assessed in this study in addition to the a priori assumed effects of body weight and age on PK parameters. The potential covariate relationships were assessed by plotting the mean a posteriori values of the PK parameters against the available covariates (weight, age, sex, dose, infusion duration, and PRISM) to identify their potential effects. If any relationship was found, it was described by means of linear regression or power model (allometric relationship). The categorical covariates were included into the model based on indicator variables.

**Table 2:** Demographic characteristic of patients

Parameter, unit	Median [range] or number
Age, months	70 [1.4–188.6]
Weight, kg	18.5 [4.7–60]
Dose, µg	1153.8 [248.8–4732.2]
Infusion duration, h	97.3 [45.0–229.2]
Pediatric risk of mortality (PRISM)	0.5 [0–11]
Male/female	23/15

Additionally the difference in PK parameters between the two occasions was tested during the model building process. The fraction parameters for all PK parameters were assumed to be equal to 1 (0 on a log scale) with precision

13 $$\log f_{P} \sim N(\log 1,\sigma_{f_{P}}^{ - 2} )$$

The selected values of *σ*_*fP*_ which spanned within a range from 0.01 to 0.6, were compared. The smallest value corresponds to a priori assumption of the lack of difference between the two occasions, and the latter one corresponds to a vague prior on the fraction parameter. The models with the lowest deviance information criterion, the best predictive performance, and the most conservative (with the lowest *σ*_*fP*_), were selected as final. A clinically significant difference in the fraction parameters was claimed when 20 % difference was observed.

### Posterior predictive check and model assessment

The model performance was assessed by means of a posteriori predictive check. The plots were drawn from individual a posteriori PK predictions. In this study the 10th, 50th and 90th percentiles were used to summarize the data and model predictions. This graph resembles the classical visual predictive check (VPC) and enables the comparison between confidence intervals obtained from prediction and the observed data over time. When the corresponding percentile from the observed data falls outside the 95 % confidence interval derived from predictions, there is an evidence of model misspecification. Since the PK data deviated from nominal times to some extent, binning across time was performed. Next, the prediction error (PE) was calculated for each measurement as PE = 100 (measured − population predicted)/population predicted, and was summarized as a median for each individual. The median prediction error (MDPE) and median absolute prediction error (MDAPE) were calculated according to the formulas:

14 $$\begin{aligned} {\text{MDPE}} = {\text{median}}(PE_{1} ,PE_{2} , \ldots PE_{n} ) \hfill \\ {\text{MDAPE}} = {\text{median}}(\left| {PE_{1} } \right|,\left| {PE_{2} } \right|, \ldots \left| {PE_{n} } \right|) \hfill \\ \end{aligned}$$

where *n* denotes the number of individuals. MDPE reflects the bias of the model, whereas MDAPE reflects the inaccuracy of the prediction.

## Results

**Fig. 1:**
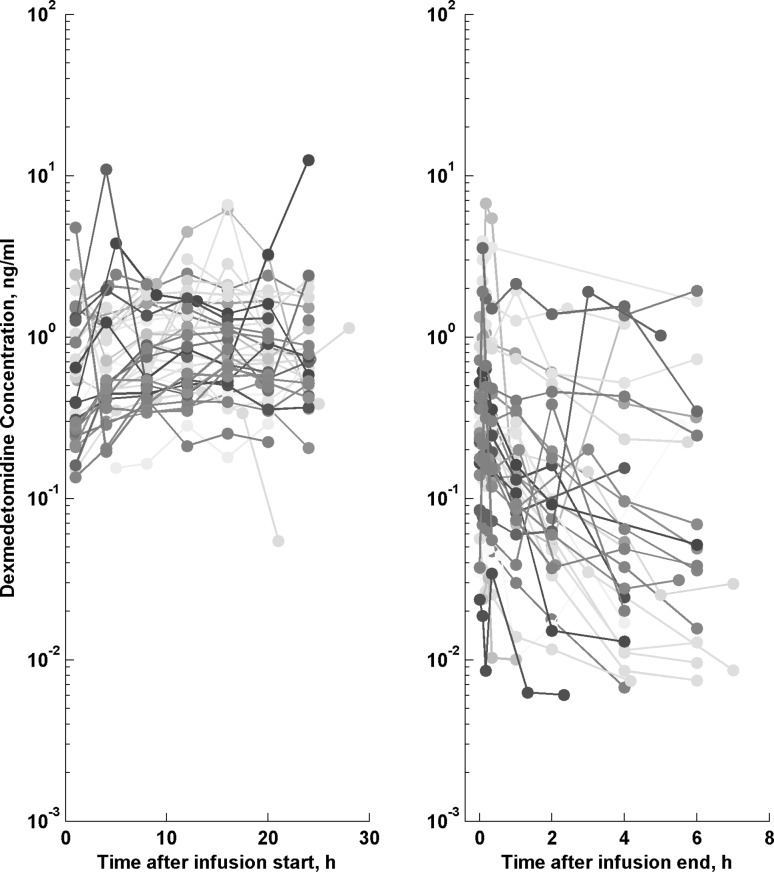
The individual dexmedetomidine concentration–time profiles

This analysis was based on the concentration–time profiles of DEX collected from 38 PICU children. Thirty-eight patients, with median (range) age of 70 months (1.4–188.6) diagnosed in our PICU with acute respiratory failure (n = 18), severe sepsis or septic shock (n = 10), multiple or brain trauma (n = 8) and acute cardiac insufficiency (n = 2), were enrolled in the study. Table 2 lists the patients’ demographic, clinical laboratory and vital signs characteristics. The available data consisted of 470 DEX concentration measurements measured at two occasions as presented in Fig. 1. In our data few outlying measurements were evident (with concentrations a few-fold higher or lower than average) and so, a robust residual error model was needed. We decided to use a t-distribution on a log-transformed concentrations.

The modeling was done using Bayesian inference with informative priors. It was required due to small number of patients and observational nature of the study design, which limited the ability to precisely estimate all PK parameters. The model-building process started with a two-compartment model, for which after implementation of an allometric scaling to all clearance and volume of distribution parameters, age maturation of clearance turned out to be insufficient to describe our data. Without occasion as a covariate there was an evidence of miss-prediction in the initial phase after infusion cessation as reflected by MDPE (−11.50) and posterior predictive check (data not shown). The inclusion of fractional change for all PK parameters improved the model as demonstrated by lower DIC value (DIC changed from 840.86 to 825.68, ΔDIC = 15.176) and reduced the bias (MDPE decreased from −11.50 to −2.2) observed initially in predictive check plots. The supplementary materials present the influence of prior precision on the posterior distribution of parameters. The *σ*_*fP*_ = 0.2 represents the most parsimonious choice as further increase in its values did not improve the accuracy of model predictions.

**Fig. 2:**
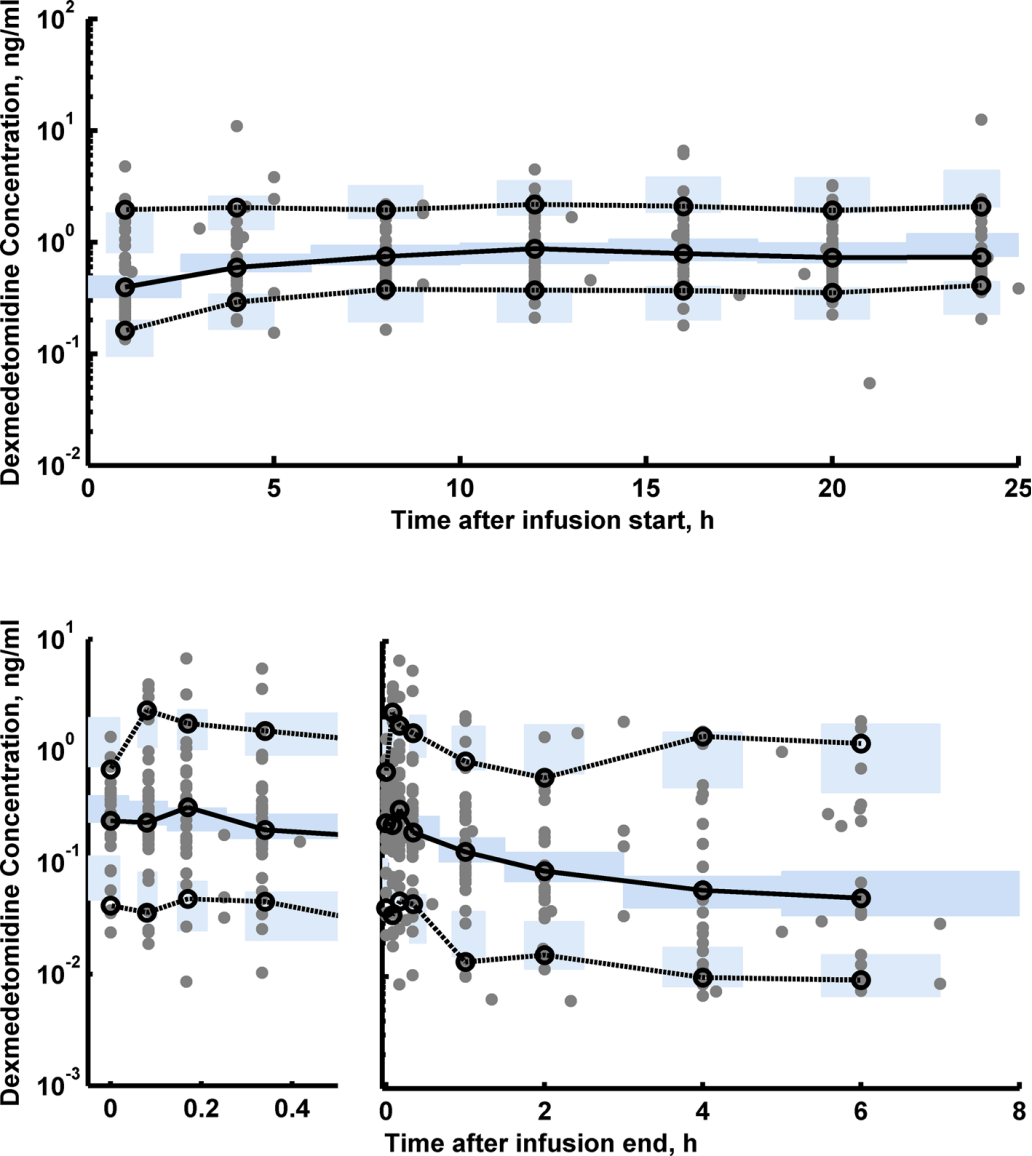
The visual a posteriori predictive plots for final dexmedetomidine PK model. The plots show the individual prediction-based 95 % confidence intervals around the 10th, 50th, and 90th percentiles of the PK data (*blue areas*). The corresponding percentiles from the experimental data are plotted in *black color*. The raw data is presented as *gray closed symbol* (Color figure online)

The goodness-of-fit plots of the final PK model are shown in supplemental materials. The individual predictions are very close to that obtained from the experimental data, indicating good performance of the model, which is also confirmed by other goodness-of-fit diagnostic plots. The posterior predictive check for the DEX concentration was used to assess the simulation properties of the model presented in Fig. 2. Both the central tendency of the data and the variability at a particular sampling time were recaptured well. There are no major misspecifications in that graph.

Table 3 provides the means and credibility interval for all PK parameters. The typical value of the volume of the central compartment (*V*_1_) scaled to 70 kg was 52.0 L, whereas the volume of the peripheral compartment was slightly higher (*V*_2_ = 70.4 L). The typical systemic clearance (*CL*) of DEX and the distribution clearance (*Q*) were 41.6 and 56.8 L/h for 70-kg patients. The IIV was estimated for the *CL, Q*, and *V*_1_ and *V*_2_, for which it amounted to 56, 83, 152, 68 % and a strong correlation (0.7) between *Q* and *V*_1_. Those values are consistent with literature parameters in children and adults and are very close to the priors used [34]. The change between mean prior and mean posterior values was −1.2, −27.5, −7.6, 2.0, −4.5, −4.3 for *CL*, *Q*, *V*_1_, *V*_2_, *TE*_50_ and Hill, respectively.

**Table 3:** Summary of the MCMC simulations of the marginal posterior distributions of pharmacokinetic parameters from the final model of dexmedetomidine

Parameter, unit	Description	*θ*, Mean (90 % HDI)
*θ*_CL_, L/h 70 kg^−1^	Total clearance	41.6 (39.0–44.3)
*θ*_Q_, L/h 70 kg^−1^	Distribution clearance	56.8 (43.5–73.5)
*θ*_V1_, L 70 kg^−1^	Volume of distribution of central compartment	52.0 (43.2–59.6)
*θ*_V2_,L 70 kg^−1^	Volume of distribution of peripheral compartment	70.4 (63.0–79.8)
*θ*_TE50_, weeks	Age at which clearance is 50 % of adult value	42.5 (61.7–47.8)
*θ*_Hill_	Slope of clearance maturation	2.45 (1.72–3.39)
*f*_CL_	Fractional change of CL	1.31(0.910–1.82)
*f*_Q_	Fractional change of Q	1.02 (0.722–1.40)
*f*_V1_	Fractional change of V1	1.50 (1.33–1.65)
*f*_V2_	Fractional change of V2	0.86 (0.630–1.17)

*HDI* highest density interval

The final model included the difference in PK parameters between two occasions as reflected by the fraction parameters *f*_*P*_. The posterior probability for inclusion of the fractional effect on occasion was >0.5 for a 20 % change in the parameters (Pr = 0.62 for *CL* and Pr = 1.00 for *V*_1_). For other parameters the probability was lower than 0.5. The volume of distribution and clearance was 1.5-fold (with 5th–95th credible interval of 1.33–1.65) and 1.3-fold (with 5th–95th credible interval of 0.91–1.82) higher at the second occasion, respectively.

**Fig. 3:**
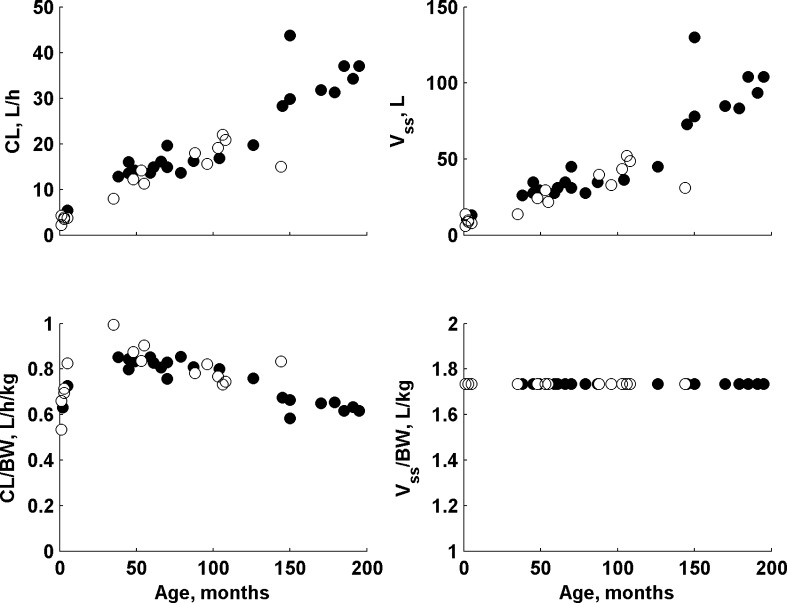
The individual mean a posteriori values of clearance and volume of distribution (actual and normalized by body weight) in relation to patient age. *Closed symbols* denote males and *open symbols* denote females

The children enrolled in this study exhibited a large difference in body weight ranging from 4.7 to 60 kg. In this study the allometric scaling with theory-based exponents for all clearance and volume terms was used along with a clearance maturation model. The actual and body weighted normalized values of clearance and volume of distribution in relation to patient age are presented in Fig. 3. None of the covariate (Table 2) was found to be statistically significant in this study (in addition to the a priori assumed age and body weight effects) as there is no clear relationship between them and individual PK parameter estimates. The ETA plots (deviation of the individual estimate from the population mean in relation to covariate) for age, duration of infusion, PRISM, sex, weight are shown in supplementary materials.

## Discussion

A population pharmacokinetic model was developed based on the data obtained from PICU in critically ill patients undergoing prolonged infusion. The available small group of patients with wide age and weight range and potential outliers enforced the use of a fully Bayesian approach with informative prior information on the PK parameters to increase the stability of the model developed. The priors were elucidated from one study involving a pooled analysis of various DEX data obtained in children [34].

An interesting phenomenon from the point of view of the pharmacokinetic properties of DEX is increasing clearance during infusion of the drug reported in critically ill adult patients in the ICU. This observation was noted in a study by Iirola et al. [5], in which 13 critically ill patients were treated with constant infusion rate of DEX for the first 12 h. After the first 12 h, the infusion rate of DEX was titrated between 0.1 and 2.5 mg/kg/h by using a predefined dose levels to maintain sedation within the range between 0 and 3 on the Richmond Agitation-Scale Sedation. DEX infusion was continued as long as required to a maximum of 14 days. The authors explain this more than two-fold increase in drug clearance by general improvement in the physiological condition of the patients and improvement in liver flows [5]. Our data show moderate evidence of a clinically significant increase in clearance (Pr = 0.62) at the second (post infusion end) occasion. Addressing the literature observation in adults, it is reasonable to conclude that this phenomenon is likely to occur in children.

There is also a strong evidence of an increased volume of the central compartment after infusion cessation. That is very likely due to an unavoidable phenomenon that during the routine administration of the drug, there is a moment when the drug enters the bloodstream despite the end of infusion, as a consequence of its presence in the drug delivery lines. Thus, we observe higher DEX concentrations than expected which in consequence leads to increased value of *V*_1_. This increased value of *V*_1_ translates to the increased half-life of the alpha-phase and decreased elimination rate of DEX few minutes upon infusion discontinuation. Also other explanations cannot be excluded, such as altered cardiac index during the recovery after anesthesia, which can alter perfusion rates to tissues and leads to higher *V*_1_ estimates [35, 36].

In this work, the presence of outliers in the data was handled by assuming a robust t-distribution of residuals. Our approach was different from the one already presented for DEX [37]. In the cited work authors used a finite mixture as the residual error model. Nevertheless, this particular approach could not be used for our dataset as it required unrealistic assumption of an additive residual error model and one compartment disposition model.

The personalized therapy requires the knowledge of drugs pharmacokinetics and factors affecting inter-individual variability in drug response [38]. The elucidation of those factors seems to be particularly important in the pediatric population treated in PICU [39]. We think that the use of Bayesian inference approach might be an effective tool in addressing the often asked questions on the most likely differences between the population of patients investigated and the one that was used to support the current dosing paradigm. This post-data questions are often present when analyzing observational data and can effectively be addressed using Bayesian theory.

In conclusion, a population PK model was successfully developed to describe the time course and variability of dexmedetomidine in PICU patients using allometric principles and clearance maturation model. The disease status described by PRISM score, duration of infusion, and sex were not found to be independent significant covariates in this study. A 1.5-fold increase in the volume of distribution after infusion cessation was observed. There were also some evidences on increased clearance, however, more data is needed to fully confirm clinical significance of this phenomenon.

Supplementary material is available and includes mass spectrometry settings, trace plots of model parameters along the MCMC chain’s length, details on selection of fraction parameters (*f*_*P*_), goodness-of-fit plots, ETA plots, and WinBUGS/Matlab codes of the used models.

## Electronic supplementary material

Below is the link to the electronic supplementary material.

Supplementary material 1 (DOCX 1830 kb)
