# Supplementary material for: The pharmacokinetics of dexmedetomidine during long-term infusion in critically ill pediatric patients. A Bayesian approach with informative priors
Source: J Pharmacokinet Pharmacodyn. 2016 May 24;43:315–24. doi: 10.1007/s10928-016-9474-0 (PMC4886153; doi:10.1007/s10928-016-9474-0)
Supplement: Supplementary file 1 — Supplementary material 1 (DOCX 1830 kb) [file 10928_2016_9474_MOESM1_ESM.docx]

# Supporting Online Material to “The pharmacokinetics of dexmedetomidine during long-term infusion in critically ill pediatric patients. A Bayesian approach with informative priors.”

Paweł Wiczling, Alicja Bartkowska-Śniatkowska, Oliwia Szerkus, Danuta Siluk, Jowita Rosada-Kurasińska, Justyna Warzybok, Agnieszka Borsuk, Roman Kaliszan, Edmund Grześkowiak , Agnieszka Bienert

Email: asniatko@umed.poznan.pl

Content:

1. Table 1S. Mass spectrometry parameters used in the study.
2. Table 2S. Summary of the MCMC simulations of the marginal posterior distributions of pharmacokinetic parameters from the final model of dexmedetomidine.
3. Figure 1S. Trace plots of model parameters along the MCMC chain’s length.
4. Figure 2S. Trace plots of variance-covariance parameters along the MCMC chain’s length.
5. Figure 3S. The effect of prior precision ($\sigma_{fP}^{-2}$) on the posterior distribution of fraction effects (*f_P_*) corresponding to particular PK parameters. The posterior distributions are summarized as median, quartiles, and 5^th^-95^th^ intervals.
6. Figure 4S. The influence of prior precision ($\sigma_{fP}^{-2}$) on the predictive properties of the DEX PK models as reflected by the bias (MDPE) and inaccuracy (MDAPE) of the model. The *σ_fP_* = 0.2 is the most conservative choice for the prior uncertainty leading to the highest predictive accuracy.
7. Figure 5S. Goodness-of-fit plots for dexmedetomidine PK.
8. Figure 6S. The individual mean *a posteriori* values of eta (deviation of the individual estimate from the population mean) of dexmedetomidine clearance and volume of distribution parameters in relation to the individual values of body weight, age, duration of infusion and PRISM scale.
9. Figure 7S. The individual mean *a posteriori* values of eta (deviation of the individual estimate from the population mean) of dexmedetomidine clearance and volume of distribution parameters in relation to sex.
10. Matlab and Winbugs Codes

Table 1S. Mass spectrometry parameters used in the study.

| **Parameter, unit** | **Value** |
| --- | --- |
| Fragmentor (V) | 95 (DEX)  105 (IS) |
| Collision energy (V) | 16 (DEX, quantifier)  36 (DEX, qualifier)  20 (IS) |
| **Ionization source parameters, unit** | |
| Gas flow (L/min) | 12 |
| Nebulizer pressure (psi) | 45 |
| Drying gas temperature (°C) | 330 |
| Capillary voltage (V) | 2000 |

Table 2S. Summary of the MCMC simulations of the marginal posterior distributions of pharmacokinetic parameters from the final model of dexmedetomidine. For covariance and correlation matrix the lower-triangular elements are provided.

| **Between Subject Variability, Mean, (90% Highest Density Interval, HDI)** | | |
| --- | --- | --- |
| **Ω**  **Corr** | Covariance Matrix  Correlation Matrix | $\left[\begin{matrix} \text{0.272(0.186}-\text{0.400)} & & & \\ \text{-0.018(-0.145}-\text{0.109)} & \text{0.527(0.269}-\text{0.889)} & & \\ \text{-0.003(-0.193}-\text{0.017)} & \text{0.543 (0.239}-\text{0.904)} & \text{1.200(0.733}-\text{1.82)} & \\ \text{-0.047(-0.193}-\text{0.086)} & \text{0.152 (-0.003}-\text{0.333)} & \text{0.211 (-0.017}-\text{0.445)} & \text{0.383(0.228}-\text{0.608)} \end{matrix}\right]$ $\left[\begin{matrix} 1 & & & \\ \text{-0.047(-0.386}-\text{0.268)} & 1 & & \\ \text{-0.006(-0.324}-\text{0.292)} & \text{0.682 (0.445}-\text{0.830)} & 1 & \\ \text{-0.145(-0.503}-\text{0.286)} & \text{0.340 (-0.008}-\text{0.613)} & \text{0.312 (-0.025}-\text{0.587)} & \text{ 1} \end{matrix}\right]$   |
| **Residual Error Model, Mean (90% Highest Density Interval, HDI)** | | |
| σ_C_ | Scale parameter of t distribution | 0.275 (0.233 – 0.317) |
| ν | Normality of t distribution | 1.75 (1.42 – 2.14) |


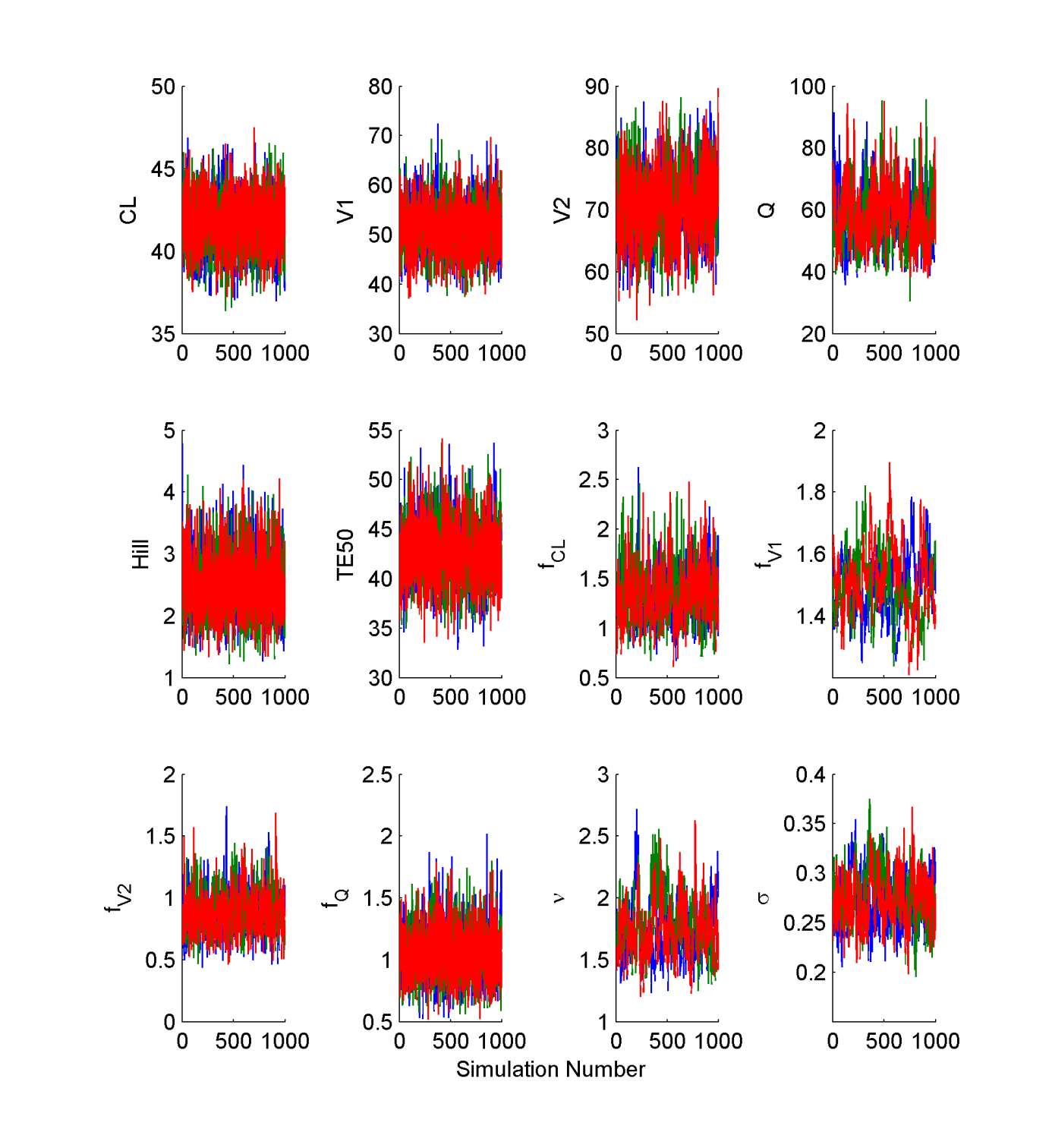


Figure 1S. Trace plots of model parameters along the MCMC chain’s length.


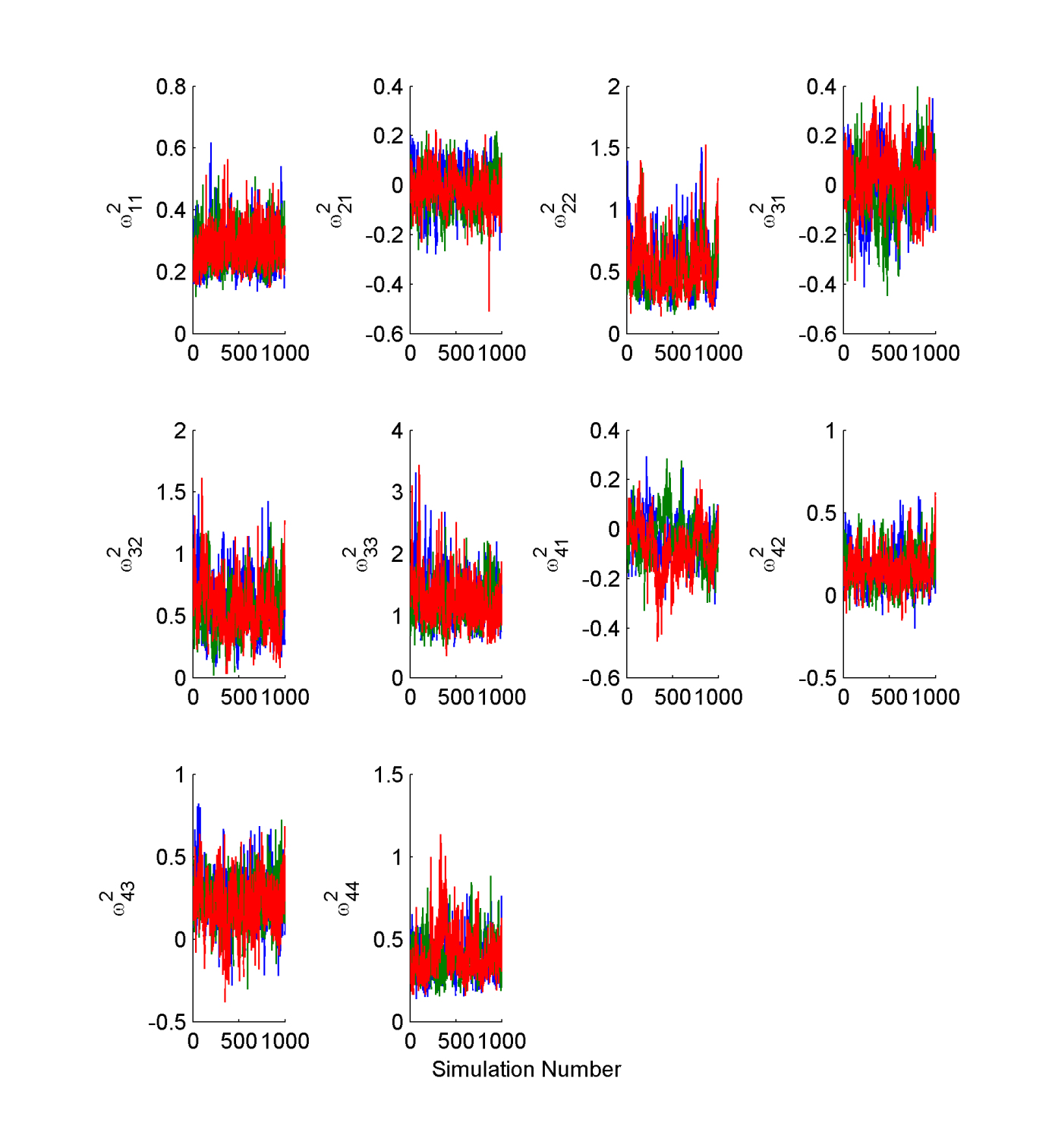


Figure 2S. Trace plots of variance-covariance parameters along the MCMC chain’s length.


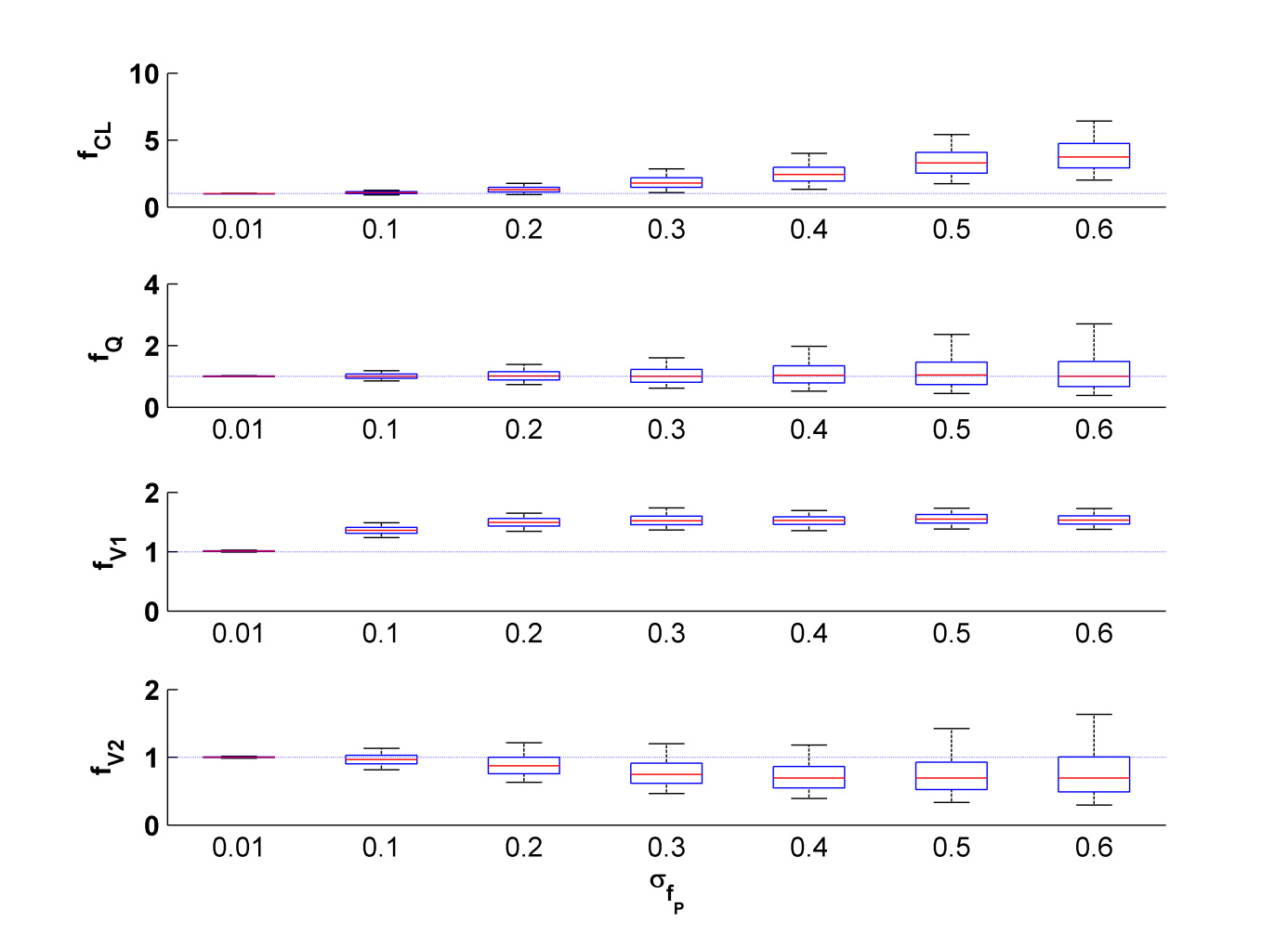


Figure 3S. The effect of prior precision ($\sigma_{fP}^{-2}$) on the posterior distribution of fraction effects (*f_P_*) corresponding to particular PK parameters. The posterior distributions are summarized as median, quartiles, and 5^th^-95^th^ intervals.


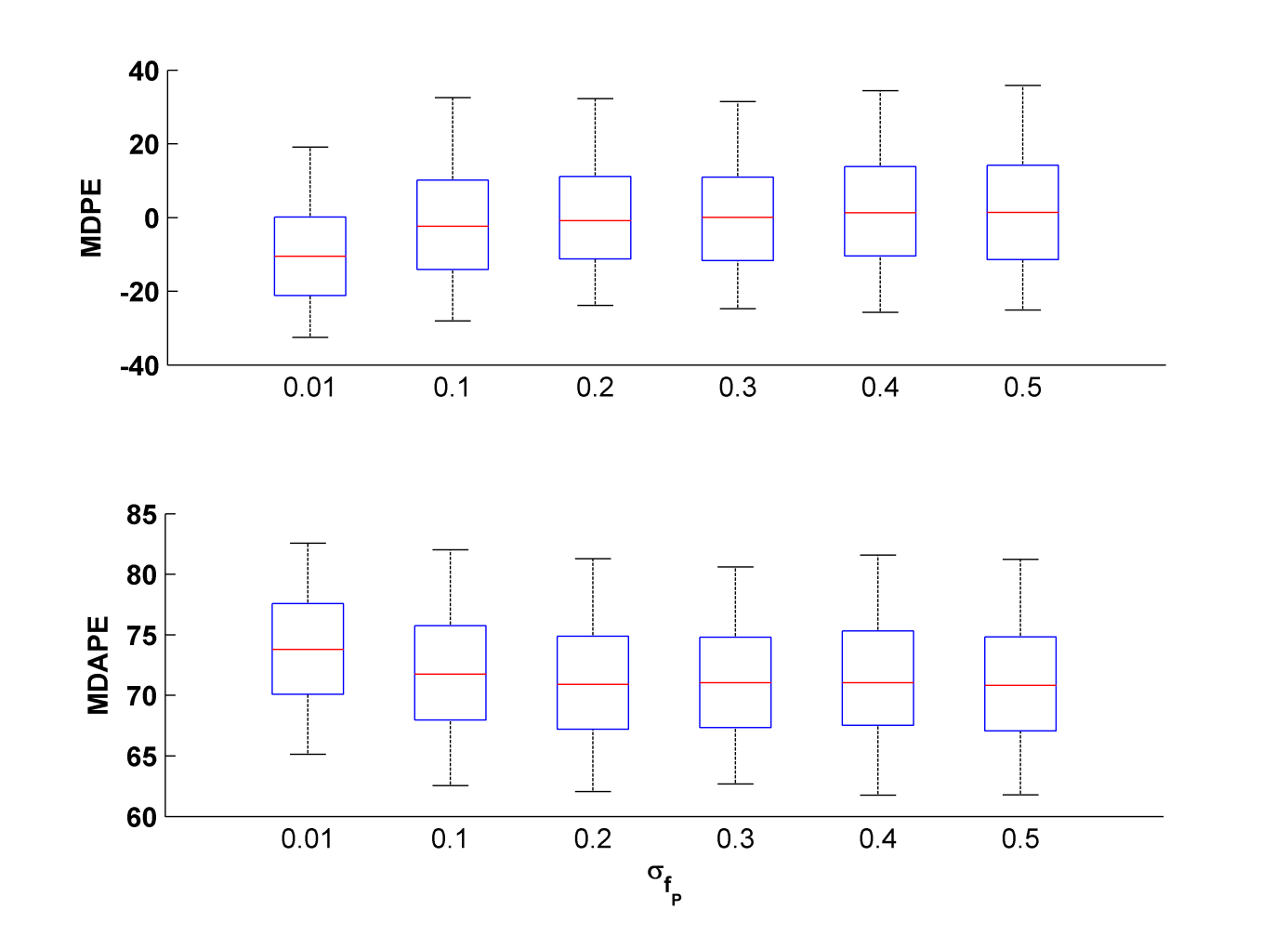


Figure 4S. The influence of prior precision ($\sigma_{fP}^{-2}$) on the predictive properties of the DEX PK models as reflected by the bias (MDPE) and inaccuracy (MDAPE) of the model. The *σ_fP_* = 0.2 is the most conservative choice for the prior uncertainty leading to the highest predictive accuracy.


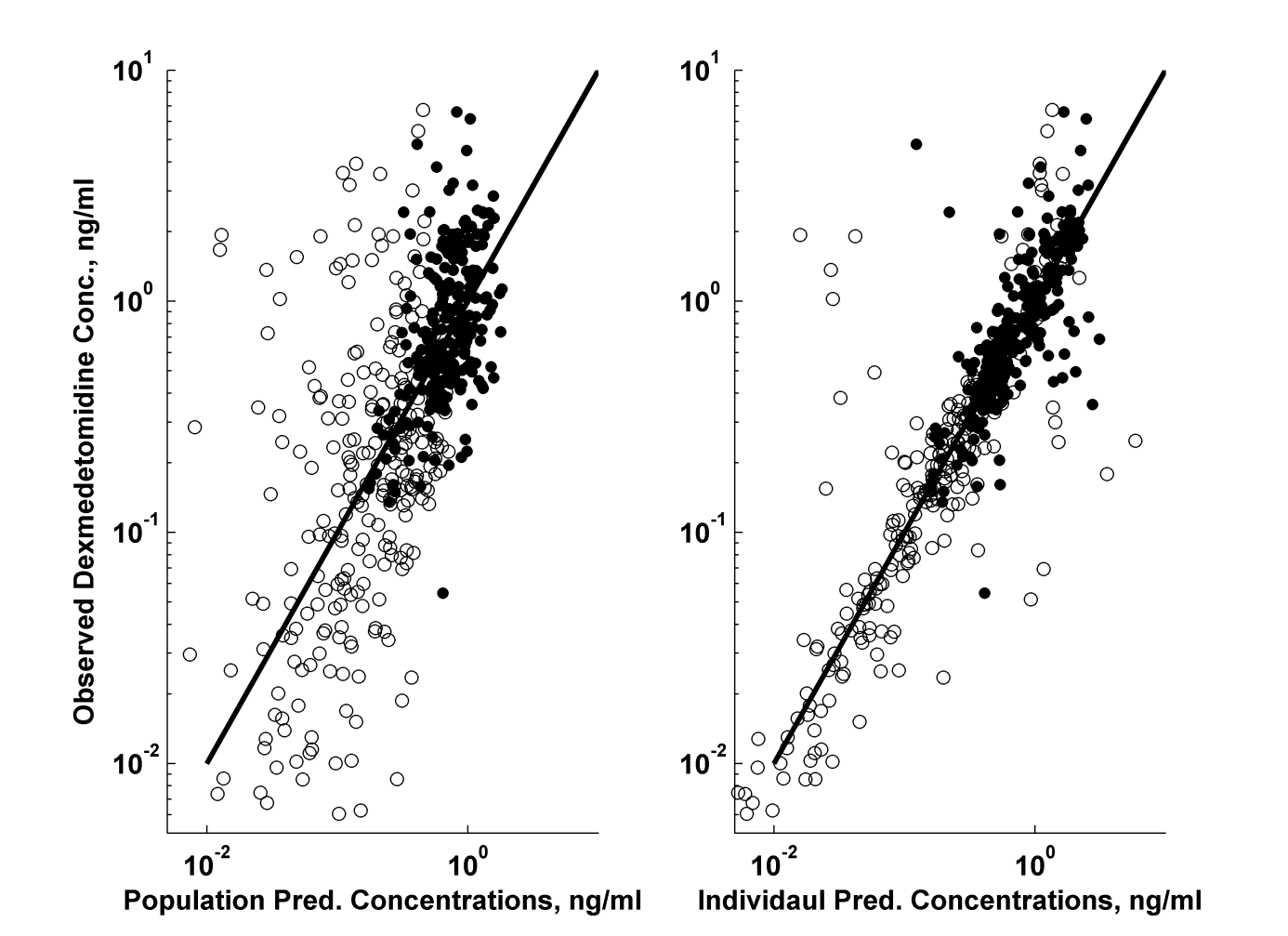


Figure 5S. Goodness-of-fit plots for dexmedetomidine PK: the observed *versus* the population predicted concentrations; the observed *versus* the individual predicted concentrations. The filled circles denote observed/predicted concentrations during the infusion duration, the empty circles corresponds to measurements/predictions after infusion cessation.


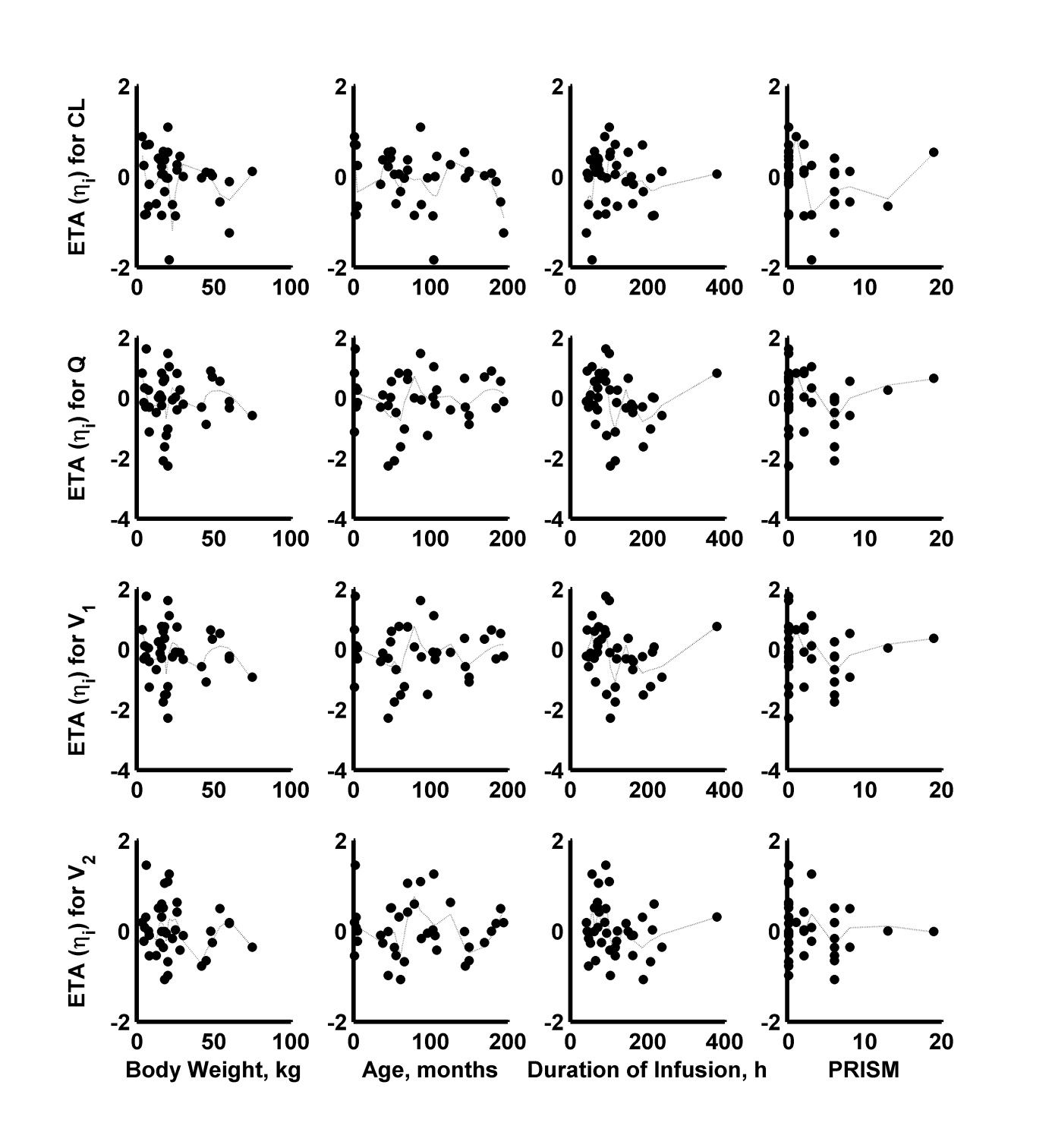


Figure 6S. The individual mean *a posteriori* values of eta (deviation of the individual estimate from the population mean) of dexmedetomidine clearance and volume of distribution parameters in relation to the individual values of body weight, age, duration of infusion and PRISM scale. The dotted line indicates the trend in the data (loess smooth).


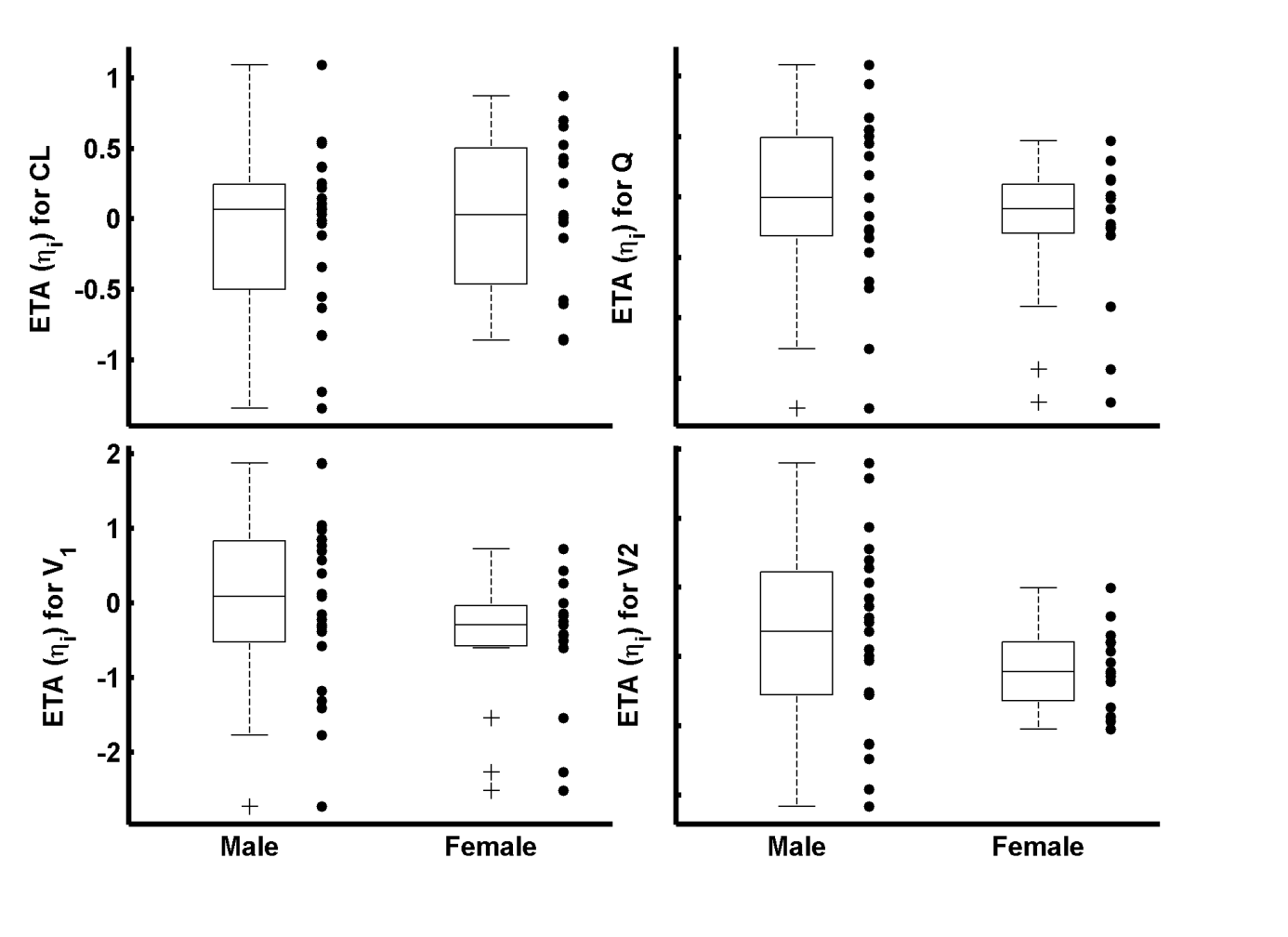


Figure 7S. The individual mean *a posteriori* values of eta (deviation of the individual estimate from the population mean) of dexmedetomidine clearance and volume of distribution parameters in relation to sex.

**The Matlab Code**

% WINBUGS Parameters

nchains = 3; % How Many Chains?

nburnin = 1000; % How Many Burn-in Samples?

nsamples = 1000; % How Many Recorded Samples?

nobs = length(time); % Number of observations

nsub = length(unique(subject)); % Number of Subjects

[k,i1,j]=unique(subject,'first'); % The index of first measurement for a particular subject

[k,i2,j]=unique(subject,'last'); % The index of last measurement for a particular subject

datastruct = struct(...

'nobs',nobs', ...

'nsub', nsub', ...

'start',i1', ...

'end',i2', ...

'subject',j',...

'weight',weight',...

'age',age',...

'time',time',...

'amt',amt',...

'rate',rate',...

'ii',zeros(nobs,1)',...

'evid',evid',...

'cmt', ones(nobs,1)',...

'addl',zeros(nobs,1)',...

'ss', zeros(nobs,1)',...

'logCobs', log(cobs)',...

'omega_inv_prior',30*diag([0.091,0.130,0.320,0.200],0),...

'tau',100); % The data for winbugs

% Initialize the values for each latent variable in each chain

for i=1:nchains

S.logCLHat = normrnd(log(45),0.2,1);

S.logQHat = normrnd(log(78),0.2,1);

S.logV1Hat = normrnd(log(56),0.2,1);

S.logV2Hat = normrnd(log(69),0.2,1);

S.logCLHatFr = normrnd(log(1),0.1,1);

S.logQHatFr = normrnd(log(1),0.1,1);

S.logV1HatFr = normrnd(log(1),0.1,1);

S.logV2HatFr = normrnd(log(1),0.1,1);

S.logTE50Hat = normrnd(log(45),0.2,1);

S.logNNHat = normrnd(log(2.7),0.2,1);

S.numone = unifrnd(1,5,1);

S.omega_inv = inv(diag((diag(exp(2*normrnd(log(0.25),0.5,4)))),0));

S.sigmaC = unifrnd(0.1,2,1);

init0(i) = S;

end

%% Use WinBUGS to Sample through the MatBUGS interface

fprintf( 'Running WinBUGS...\n' );

tic

[samples0, stats0] = matbugs(datastruct, ...

fullfile(pwd, '200DexModelTdistrIinfprioCLT.txt'), ...

'init', init0, ...

'nChains', nchains, ...

'view', 0, 'nburnin', nburnin, 'nsamples', nsamples, ...

'thin', 3, 'DICstatus', 1, 'refreshrate',100, ...

'monitorParams',{'CLHat', 'QHat' ,'V1Hat', 'V2Hat', 'CLHatFr', 'QHatFr', 'V1HatFr','V2HatFr', 'TE50Hat','NNHat','omega', 'sigmaC', 'nu', 'logCobsCond','logCobsPred','theta'}, ...

'Bugdir', 'C:\Program Files\BlackBoxWinBUGS');

toc

**The Winbugs Code:**

model

{

for(i in 1:nsub){

logtheta[i, 1:4] ~ dmnorm(logthetaMean[i, 1:4], omega.inv[1:4, 1:4])

logthetaMean[i, 1] <- logCLHat + 0.75*log(weight[start[i]]/70) + log(pow(4*age[start[i]]+40,NNHat)/(pow(4*age[start[i]]+40,NNHat) + pow(TE50Hat,NNHat))) # CL

logthetaMean[i, 2] <- logQHat + 0.75*log(weight[start[i]]/70) # Q

logthetaMean[i, 3] <- logV1Hat + log(weight[start[i]]/70) # V1

logthetaMean[i, 4] <- logV2Hat + log(weight[start[i]]/70) # V2

log(theta[i,5]) <- logCLHatFr + logtheta[i,1]

log(theta[i,6]) <- logQHatFr + logtheta[i,2]

log(theta[i,7]) <- logV1HatFr + logtheta[i,3]

log(theta[i,8]) <- logV2HatFr + logtheta[i,4]

theta[i,9] <- 1 # nnn

theta[i,10] <- 30# tcl50

theta[i,11] <- 1 # F1

theta[i,12] <- 1 # F2

theta[i,13] <- 0 # tlag1

theta[i,14] <- 0 # tlag2

logthetaPred[i, 1:4] ~ dmnorm(logthetaMean[i, 1:4], omega.inv[1:4, 1:4])

log(thetaPred[i,5]) <- logCLHat2 + logthetaPred[i,1]

log(thetaPred[i,6]) <- logQHat2+ logthetaPred[i,2]

log(thetaPred[i,7]) <- logV1Hat2 + logthetaPred[i,3]

log(thetaPred[i,8]) <- logV2Hat2 + logthetaPred[i,4]

thetaPred[i,9] <- 1 # nnn

thetaPred[i,10] <- 30 # tcl50

thetaPred[i,11] <- 1 # F1

thetaPred[i,12] <- 1 # F2

thetaPred[i,13] <- 0 # tlag1

thetaPred[i,14] <- 0 # tlag2

for(j in 1:4){

log(theta[i,j]) <- logtheta[i,j]

log(thetaPred[i,j]) <- logthetaPred[i,j]

}

xhat[start[i]:end[i],1:2] <- DexTwoCptModelTCLRK45OCC(time[start[i]:end[i]], amt[start[i]:end[i]], rate[start[i]:end[i]],ii[start[i]:end[i]], evid[start[i]:end[i]], cmt[start[i]:end[i]], addl[start[i]:end[i]], ss[start[i]:end[i]], theta[i,])

xhatPred[start[i]:end[i],1:2] <- DexTwoCptModelTCLRK45OCC(time[start[i]:end[i]], amt[start[i]:end[i]], rate[start[i]:end[i]], ii[start[i]:end[i]], evid[start[i]:end[i]], cmt[start[i]:end[i]], addl[start[i]:end[i]], ss[start[i]:end[i]], thetaPred[i,])

}

for(i in 1:nobs){

logCobs[i] ~ dt(logCHat[i],tauC,nu)

logCobsCond[i] ~ dt(logCHat[i],tauC,nu)

Vc[i] <- theta[subject[i],7]+(theta[subject[i],3]-theta[subject[i],7])/(1+exp(time[i]-30));

CHat[i] <- xhat[i,1]/Vc[i]

logCHat[i] <- log(max(CHat[i],eps))

logCobsPred[i] ~ dt(logCHatPred[i],tauC,nu)

VcPred[i] <- thetaPred[subject[i],7]+(thetaPred[subject[i],3]-thetaPred[subject[i],7])/(1+exp(time[i]-30));

CHatPred[i] <- xhatPred[i,1]/VcPred[i]

logCHatPred[i] <- log(max(CHatPred[i],eps))

}

# informative priors

logCLHat ~ dnorm(3.740048,541)

logQHat ~ dnorm(4.360548,34)

logV1Hat ~ dnorm(4.030695,89)

logV2Hat ~ dnorm(4.234107,138)

logCLHat2 ~ dnorm(0,tau)

logQHat2 ~ dnorm(0,tau)

logV1Hat2 ~ dnorm(0,tau)

logV2Hat2 ~ dnorm(0,tau)

logTE50Hat ~ dnorm(3.76,157)

logNNHat ~ dnorm(0.91,22.3)

log(CLHat) <- logCLHat

log(QHat) <- logQHat

log(V1Hat) <- logV1Hat

log(V2Hat) <- logV2Hat

log(TE50Hat) <- logTE50Hat

log(NNHat) <- logNNHat

log(CLHatFr) <- logCLHatFr

log(QHatFr) <- logQHatFr

log(V1HatFr) <- logV1HatFr

log(V2HatFr) <- logV2HatFr

numone ~ dexp(lambda)

lambda <- 1/10

nu <- numone + 1

tauC <- 1/(sigmaC*sigmaC)

sigmaC ~ dunif(0.001,1000)

omega.inv[1:4, 1:4] ~ dwish(omega.inv.prior[1:4, 1:4], 30)

omega[1:4, 1:4] <- inverse(omega.inv[1:4, 1:4])

eps <- 1.0E-6

**The Component Pascal model library component for DexTwoCptModelTCLRK45OCC ODE solver**

PROCEDURE **UserDerivatives**(IN theta, x: ARRAY OF REAL;

numEq: INTEGER; t: REAL; OUT dxdt: ARRAY OF REAL) ;

VAR

CL, Q, V2, V3, k10, k12, k21, CL2, NNN, TCL50, clt, qt, v2t, v3t, Q2, V22, V32: REAL;

BEGIN

CL := theta[0];

Q := theta[1];

V2 := theta[2];

V3 := theta[3];

CL2:=theta[4];

Q2:=theta[5];

V22:=theta[6];

V32:=theta[7];

NNN:=theta[8];

TCL50:=theta[9];

ASSERT((CL > 0) & (Q > 0) & (V2 > 0) & (V3 > 0)& (NNN > 0)& ( TCL50 > 0) , 20);

ASSERT((CL2 > 0) & (Q2 > 0) & (V22 > 0) & (V32 > 0), 20);

clt:=CL2+(CL-CL2)/(1+Math.Exp(NNN*(t-TCL50)));

qt:=Q2+(Q-Q2)/(1+Math.Exp(NNN*(t-TCL50)));

v2t:=V22+(V2-V22)/(1+Math.Exp(NNN*(t-TCL50)));

v3t:=V32+(V3-V32)/(1+Math.Exp(NNN*(t-TCL50)));

k10 := clt/v2t;

k12 := qt/v2t;

k21 := qt/v3t;

(* Differential equations for the model excluding piecewise constant input rates provided in the data set *)

dxdt[0] :=- (k10 + k12) * x[0] + k21 * x[1];

dxdt[1] := k12 * x[0] - k21 * x[1];
